# A new method for estimating the demographic history from DNA sequences: an importance sampling approach

**DOI:** 10.3389/fgene.2015.00259

**Published:** 2015-08-07

**Authors:** Sadoune Ait Kaci Azzou, Fabrice Larribe, Sorana Froda

**Affiliations:** Département de Mathématiques, Équipe de Modélisation Stochastique Appliquée (EMOSTA), Université du Québec à MontréalMontréal, QC, Canada

**Keywords:** importance sampling, effective population size, *skywis plot*, coalescent process, serially sampled sequences

## Abstract

The effective population size over time (demographic history) can be retraced from a sample of contemporary DNA sequences. In this paper, we propose a novel methodology based on importance sampling (IS) for exploring such demographic histories. Our starting point is the *generalized skyline plot* with the main difference being that our procedure, *skywis plot*, uses a large number of genealogies. The information provided by these genealogies is combined according to the IS weights. Thus, we compute a weighted average of the effective population sizes on specific time intervals (epochs), where the genealogies that agree more with the data are given more weight. We illustrate by a simulation study that the *skywis plot* correctly reconstructs the recent demographic history under the scenarios most commonly considered in the literature. In particular, our method can capture a change point in the effective population size, and its overall performance is comparable with the one of the *bayesian skyline plot*. We also introduce the case of serially sampled sequences and illustrate that it is possible to improve the performance of the *skywis plot* in the case of an exponential expansion of the effective population size.

## 1. Introduction

The demographic history of a population leaves its signature in the genome, which means that DNA sequences contain information about the demographic history of the population from which they are sampled. Therefore, it is possible to use genetic data to infer demographic parameters, an issue with important implications in many fields such as public health, epidemiology and conservation biology (Minin et al., 2008).

The first methods for estimating the demographic history from gene sequences were parametric and used coalescent theory. Such methods require a simple demographic model in order to describe the changes in the population size over time in terms of one or more parameters. They are based on importance sampling, e.g., (Slatkin and Hudson, 1991; Stephens and Donnelly, 2000), or Markov Chain Monte Carlo (MCMC) sampling, e.g., (Kuhner et al., 1995, 1998). For example, in the case of exponential growth, the size of the population at any time *t* measured from the present to the past is given by *N*(*t*) = *N*(0)exp(−β*t*), and the unknown parameters are *N*(0) and β.

Usually, in practice, it is not known in advance which demographic model fits the sampled gene sequences. Further, population histories are often more complex than those described by simple parametric models. This has motivated the development of non-parametric and semi-parametric methods for inferring the demographic history from sequence data or from an estimated genealogy (e.g., Fu, 1994; Pybus et al., 2000) without resorting to some previous information about the demographic model.

Our method is nonparametric and is closely related to the family of *skyline plot* methods. The first method in this family was introduced by Pybus et al. (2000), and is referred to as the *classical skyline plot*. The *classical skyline plot* involves two separate steps, see (Ho and Shapiro, 2011): (1) estimating the genealogy from the sequence data and (2) estimating the population history from the estimated genealogy. Step 1 gives an estimated genealogy that includes the relationships among the individuals (tree topology) as well as their times of divergence. Genealogical estimation is done using standard phylogenetic methods under the so-called strict molecular clock. The strict molecular clock condition means that the branch lengths of the tree are proportional to time, with time being measured in mutations, and all lineages evolve at the same rate. It is also possible to estimate a genealogy in a relaxed-clock framework (Drummond et al., 2006). Further, in step 2 in order to estimate the population history from the estimated genealogy, Pybus et al. (2000) apply coalescent theory in a specific way by considering the times of divergence (node times) as coalescent times. When the true population size is constant, this assumption is equivalent to estimating the mean of an exponential distribution using a single realization from this distribution (Minin et al., 2008). This uncertainty is referred to as coalescent error. Further, the single phylogeny of the sequences is assumed to be known without error (i.e., phylogenetic error is assumed to be negligible).

Thus, Pybus et al. (2000) estimate the population size $\hat{N}e_{k}\mu$, for each coalescent interval γ_*k*_ = μ*t*_*k*_, by the product of $\left(\begin{array}{c} k\\ 2 \end{array}\right)$ and γ_*k*_, where μ is the mutation rate per site per generation and γ_*k*_ is measured in substitutions. Thus, the *classical skyline plot* produces a piecewise reconstruction of the demographic history that is quite *noisy*, especially in the presence of small intervals when the sampled sequences are similar.

To improve the *classical skyline plot* estimation, several extensions have been proposed. Without being exhaustive, we discuss the extensions that are most relevant to our work.

Strimmer and Pybus (2001) developed a *generalized skyline plot* estimate based on the Akaike Information Criterion correction (AIC) in order to reduce the number of free parameters in the *classical skyline plot*. This method allows multiple coalescent events, i.e., for which little divergence time information is available, to be grouped together. Important developments were obtained in a Bayesian framework. Thus, Drummond et al. (2005) and Opgen-Rhein et al. (2005) use multiple change-point (MCP) models to estimate population size dynamics.

In particular, Drummond et al. (2005) use a Markov chain Monte Carlo (MCMC) sampling procedure that efficiently samples a variant of the *generalized skyline plot*, given sequence data, and combines these plots in order to generate a posterior distribution of the effective population size through time. Due to the averaging effect of the MCMC sampling, the *Bayesian skyline plot* introduced by Drummond et al. (2005) produces smoother estimates than previous skyline plot methods. Also in the Bayesian framework, Minin et al. (2008) propose an alternative to change-point modeling that exploits Gaussian Markov random fields to achieve temporal smoothing of the effective population size. The advantage of the *skyride* method is that in contrast to estimates given by MCP models, explicit temporal smoothing does not require strong prior decisions like fixing the total number of change points *a priori*.

Finally, Heled and Drummond (2008) introduced the extended Bayesian skyline plot, which permits the analysis of multiple unlinked loci. Increasing the number of independent loci allows the uncertainty in the coalescent to be assessed, leading to an improvement in the reliability of the demographic inference and a substantial reduction in estimation error (Ho and Shapiro, 2011). Further, unlike previous *skyline plot* methods that use a piecewise-constant model, the extended Bayesian skyline plot permits the use of a piecewise-linear model to describe the demographic history, allowing the population size to change continuously along each interval.

In order to estimate the effective population size, we propose a new method in a likelihood-based perspective. Unlike some skyline methods that use a single estimated phylogeny of the sequences, or others that use MCMC approaches, we resort to an efficient importance sampling scheme and our estimate comes to an weighted average over a large number of simulated genealogies, each with a different set of coalescence times. The methodology is described in detail in Section The Skywis Method.

## 2. Background

### 2.1. Coalescent theory

In this section, we present the basic ideas behind the standard coalescent, as well as its extension to the case of fluctuating population size. An introduction to coalescent theory can be found in Nordborg (2003). Coalescent theory allows one to produce genealogies relating the sampled sequences according to a large class of population genetic models. In particular, the classical coalescent process assumes a single, isolated and panmictic population (e.g., a Wright-Fisher model), which evolves with constant (haploid) size *N* over many generations. For sufficiently large *N* and a sample size *n* such that *n*≪*N*, the ancestral relationships between the gene sequences can be approximated by Kingman's coalescent (Kingman, 1982).

In short, the ancestry of a sample of sequences is modeled back in time, starting from the current sample and until the most recent common ancestor (MRCA) of the sample is found. At each step in the genealogical tree, one of the following events can occur: (1) two sequences coalesce if they share a common ancestor; (2) one sequence mutates. In the coalescent framework, time is measured in units of *N* generations, and *N* is large. The mutation rate μ per sequence per generation is rescaled so that θ = 2*Nμ*. Further, one can consider that each pair of lineages coalesces independently as a Poisson process with rate 1, and so, when there are *k* ancestral lines, coalescent events occur as in a Poisson process with total rate *k*(*k* − 1)/2 (Stephens, 2000).

In the classical coalescent process, and in the presence of *k* gene sequences, the waiting time *T*_*k*_ to the next coalescent event is exponentially distributed with rate $\left(\begin{array}{c} k\\ 2 \end{array}\right)$, while the distribution of the time until the first mutation event in any of the *k* lineages is exponential with parameter *kθ*/2. Since mutations are assumed to occur independently of coalescence, the waiting time until a mutation or coalescent event is exponentially distributed with parameter

(1) $$\left(\begin{array}{c} k\\ 2 \end{array}\right)+\frac{k\theta }{2}=\frac{k(k-1+\theta )}{2}.$$

The classical coalescent framework can be extended to include simple deviations from the idealized Wright-Fisher model, like recombination, fluctuating population size, population structure, and selection. In our paper, we focus on a single extension of the coalescent, namely variable population size.

In the case of non-constant population size, the number of descendants of a sequence in one generation does not follow the Poisson distribution with intensity one (Hein et al., 2005). As a result, when the basic coalescent is used to model a real physical population, the size *N* of the population in the (haploid) Wright-Fisher model cannot be assumed to be equal to the size of the real population.

Let *N*_*e*_(*t*) denote the effective population size at time *t* with *N*_*e*_(0) = *N*. The effective population size reflects the number of individuals that contribute offsprings to the descendant generation and is almost always smaller than the census population size. The variable population size coalescent model for contemporary gene sequences was introduced by Griffiths and Tavaré (1994c) and Donnelly and Tavaré (1995). In this case, the coalescence times *T*_2_, *T*_3_, …, *T*_*n*_ do not follow independent exponential distributions.

Let *V*_*k*_ = *T*_*n*_ + … + *T*_*k*_ be the accumulated waiting time so that the number of sequences pass from *n* to *k* − 1 sequences, i.e.,

(2) $$V_{k}=\sum_{\ell \;=\;k}^{n}T_{\ell },$$

and let Λ(*t*) the cumulative coalescent rate over time measured relative to the rate at time *t* = 0:

(3) $$\Lambda (t)=\int_{0}^{t}\frac{1}{\nu (u)}du,$$

where ν(*t*) = *N*_*e*_(*t*)/*N*, the relative size of *N*_*e*_(*t*) to *N*.

The waiting time until the next event depends only on the time of the previous event by the Markov property. The survival function of the time *T*_*k*_ conditional on *V*_*k* + 1_ = *v* is

(4) $$P(T_{k}>t|V_{k\;+\;1}=v)=\exp\left\{-\left(\begin{array}{c} k\\ 2 \end{array}\right)(\Lambda (t+v)-\Lambda (v))\right\},$$

where *v*_*n* + 1_ = 0.

We note that when replacing Λ(*t*) by *t* (i.e., in the case *N*_*e*_(*t*) = *N*, *t* > 0) in Equation (4), we get the survival function of the exponential distribution. From Equation (4), we obtain the density

(5) $$f_{T_{k}|V_{k+1}}(t_{k}|v)=\frac{\left(\begin{array}{c} k\\ 2 \end{array}\right)}{N_{e}(t_{k}+v)}\exp\left[-\int_{v}^{t_{k}\;+\;v}\frac{\left(\begin{array}{c} k\\ 2 \end{array}\right)}{N_{e}(x)}dx\right].$$

It is precisely from this equation that Pybus et al. (2000) derived the estimation of the effective population size $\hat{N}e_{k}$ in the presence of *k* sequences.

### 2.2. Importance sampling

Parameter estimation in population genetic models require optimization of the likelihood of the data given the parameters, *P*(D|θ). The likelihood is then evaluated by:

(6) $$L(\theta )=\int_{G}P(D|G,\theta )P(G|\theta )dG,$$

where θ is the collection of parameters (such as population size and migration rates) for the population process. Typically, the objective of the analysis is to estimate these parameters by averaging the likelihood over all possible genealogies. A naive Monte Carlo method for the integral in Equation (6) is given by

(7) $$L(\theta )\approx \frac{1}{J}\sum_{j\;=\;1}^{J}P(D|G^{\left(j\right)},\theta ),$$

where G^(1)^,G^(2)^, …,G^(*J*)^ are an independent sample from *P*(G|θ).

Importance Sampling (IS) allows us to improve the efficiency of the Monte Carlo integration. The main idea of the IS approach is to reduce the inefficiency of the approximation (Equation 7) by concentrating the simulation on the trees that are more likely with the observed data. Instead of choosing histories from the distribution *P*_θ_(G), we want to sample genealogies from a proposal distribution *Q*(G) that better supports the observed data, D. The IS method is based on rewriting (Equation 6) as

(8) $$\int_{G}P(D|G,\theta )\frac{P(G|\theta )}{Q(G)}Q(G)dG.$$

The Monte Carlo approximation of Equation (8) gives

(9) $$L(\theta )\approx \frac{1}{J}\sum_{j\;=\;1}^{J}P(D|G^{\left(j\right)},\theta )\frac{P(G^{\left(j\right)}|\theta )}{Q(G^{\left(j\right)})},$$

where G^(1)^,G^(2)^, …,G^(*J*)^ ~ *Q*(G). Good choices of the distribution *Q*(.) make this method of approximation much more efficient than (Equation 7). Ideally, we would like to sample from *Q*(G) = *P*(G|*D*). However, this is impossible because it supposes perfect knowledge of the likelihood which is not true in practice.

Importance sampling (IS) was first used in this context by Griffiths and Tavaré (1994a,b,c). Stephens and Donnelly (2000) proposed improvements to the method by suggesting an approximation to an optimal proposal distribution for IS, *P*(G|*D*).

## 3. The skywis method

In this section, we describe our estimation method of the effective population size, when *n* gene sequences are available. The main idea behind our method is to simulate a large number of genealogies and create a weighted average of the effective population sizes, where the most probable genealogies are given larger weight. In short, reconstructing the demographic history from these sequences involves four distinct steps:

1. simulate *J* genealogies: G^(1)^,G^(2)^, …,G^(*J*)^;
2. compute $\hat{N}e_{k}^{\left(1\right)},\hat{N}e_{k}^{\left(2\right)},\ldots ,\hat{N}e_{k}^{\left(J\right)}$ where $\hat{N}e_{k}^{\left(j\right)},k=2,3,\ldots ,n$, represents the estimated effective population size for the genealogy G^(*j*)^ for each coalescent time $t_{k}^{\left(j\right)}$ (in the presence of *k* sequences);
3. compute the weights *w*^(1)^, *w*^(2)^, …, *w*^(*J*)^, where *w*^(*j*)^ represents the weight of the genealogy G^(*j*)^ in the likelihood of the data;
4. estimate $\hat{N}e_{k}$ based on genealogies G^(1)^,G^(2)^, …,G^(*J*)^, by the weighted mean of $\hat{N}e_{k}^{\left(j\right)}$, for *j* = 1, 2, …, *J*, and *k* = 2, 3, …, *n*, i.e.,

(10) $$\hat{N}e_{k}=\sum_{j\;=\;1}^{J}w^{\left(j\right)}\hat{N}e_{k}^{\left(j\right)}.$$

For example, with a variable population size that is expanding from the past to the present, as we progress toward the MRCA one can expect the population size to be smaller, or coalescence times to be shorter, than in the case of a constant population size. This fact, of shorter coalescence times, should be reflected more faithfully by the most probable genealogies. Since such genealogies receive the largest weights, one can see that through the weighting system the estimator is adapting itself to the information contained in the data.

In what follows we describe our method in full detail, namely:

- how to simulate genealogies;
- how to set the weights;
- how to estimate the effective population size.

### 3.1. *Skywis plot* for homochronous sampling

#### 3.1.1. Simulation of genealogies

In order to generate genealogies we use the proposal distribution *Q*(.) introduced by Stephens and Donnelly (2000) assuming a constant population size and a finite sites model with known mutation parameters. Given the Stephens and Donnelly (2000) method is crucial to our approach, we describe it briefly.

**Figure 1:**
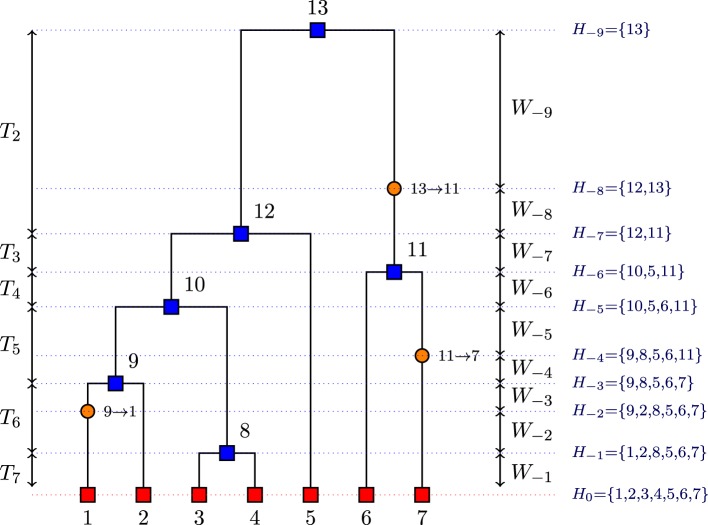
**Example of a realization of the coalescent process viewed from past to the present with *n* = 7 sequences (red squares), with 6 coalescent events (blue squares) and 3 mutation events (orange circles)**.

Let:

- *E*: the set of possible types of gene sequences;
- *H*_−*i*_: the set of all sequences when event *i* occurs (coalescence or mutation) where *i* decreases from the present to the past in steps of 1 for each event (see Figure 1);
- H = {*H*_0_, *H*_−1_, …, *H*_−*m*_}: a history of sequences where *H*_0_ = D, *m* is the total number of events in the history H, and *H*_−*m*_ is a singleton (the MRCA);
- **P**: the mutation transition matrix;

In the Stephens and Donnelly (2000) method, the *H*_−*i*_ are viewed as states of a Markov process starting at genetic type *H*_−*m*_ ∈ *E* and ending with *H*_0_ ∈ *E*. Let **P** be the mutation transition matrix. Let *P*_αβ_ be the probability of a DNA sequence of type α to mutate to a DNA sequence of type β, and let $M_{\alpha }^{\beta }$ denote a mutation of a gene sequence from type α to type β according to ***P***; let $C_{\alpha }^{\alpha }$ denote a coalescence of two gene sequences of type α. Then, the forward transition probabilities *p*_θ_(*H*_*i*_|*H*_*i*−1_), are defined by Equation (11):

(11) $$p_{\theta }(H_{i}|H_{i\;-\;1})=\{\begin{array}{cc} \frac{n_{i\;-\;1}^{\left(\alpha \right)}}{n_{i-1}}\frac{\theta }{\left(n_{i\;-\;1}-1+\theta \right)}P_{\alpha \beta } & \text{ if }M_{\alpha }^{\beta },\\ \frac{n_{i\;-\;1}^{\left(\alpha \right)}}{n_{i-1}}\frac{n_{i\;-\;1}-1}{n_{i\;-\;1}-1+\theta } & \text{ if }C_{\alpha }^{\alpha },\\ 0 & \text{otherwise,} \end{array}$$

where $n_{i-1}^{\left(\alpha \right)}$ is the number of sequences of type α in *H*_*i*−1_, *n*_*i*−1_ is the number of sequences in *H*_*i*−1_.

Stephens and Donnelly (2000) consider randomly constructing histories backward in time in a Markov way, from the sample *H*_0_ to an MRCA (single type), according to some backward transition probabilities *q*_θ_(*H*_*i*−1_|*H*_*i*_) in the class M = {*H*_*i*−1_|*P*_θ_(*H*_*i*_|*H*_*i*−1_)>0} with the constraint *q*_θ_(*H*_*i*−1_|*H*_*i*_) ∝ *p*_θ_(*H*_*i*_|*H*_*i*−1_). Their proposed backward transition probabilities $\tilde{q_{\theta }}\left(H_{i-1}|H_{i}\right)$ which define *Q*(.) are given by Equation (12), namely:

(12) $$\tilde{q_{\theta }}(H_{i\;-\;1}|H_{i})=\{\begin{array}{cc} C^{-1}\frac{\theta }{2}\cdot n_{i}^{\left(\alpha \right)}\cdot \frac{\hat{\pi }(\beta |H_{i}-\alpha )}{\hat{\pi }(\alpha |H_{i}-\alpha )}\cdot P_{\alpha \beta } & \text{ if }M_{\alpha }^{\beta },\\ C^{-1}\left(\begin{array}{c} n_{i}^{\left(\alpha \right)}\\ 2 \end{array}\right)\cdot \frac{1}{\hat{\pi }(\alpha |H_{i}-\alpha )} & \text{ if }C_{\alpha }^{\alpha },\\ 0 & \text{otherwise,} \end{array}$$

where $n_{i}^{\left(\alpha \right)}$ is the number of sequences of type α in *H*_*i*_, *n*_*i*_ is the number of sequences in *H*_*i*_, {*H*_*i*_ − α} is the set of all sequences in *H*_*i*_ without the chosen sequence α, and *C* = *n*_*i*_(*n*_*i*_ − 1 + θ) / 2 is a constant of proportionality. The estimated conditional probability $\hat{\pi }\left(\alpha |H_{i}\right)$ is described below.

In the proposed reconstruction, when *H*_*i*_ contains *n*_*i*_ chromosomes, the new type α is obtained by choosing a chromosome from *H*_*i*_ at random and then mutating it a geometric number of times. If $n_{i}^{\left(\beta \right)}$ is the number of chromosomes of type β in *H*_*i*_, then (Stephens and Donnelly, 2000),

(13) $$\hat{\pi }(\alpha |H_{i})=\sum_{\beta \in E}\sum_{m\;=\;0}^{\infty }\frac{n_{i}^{\left(\beta \right)}}{n_{i}}{\left(\frac{\theta }{n_{i}+\theta }\right)}^{m}\frac{n_{i}}{n_{i}+\theta }{\left(P^{m}\right)}_{\alpha \beta }.$$

In our approach, the genealogies are simulated backwards in time by the following algorithm based on Equation (12):

1. initialize *n*_*i*_: = *n*, where *n* is the number of DNA sequences at time *t* = 0 (present), and *i* = 0;
2. simulate the time to the next event, *W*_−*i*−1_, as an exponential distribution with parameter $\left(\begin{array}{c} n_{i}\\ 2 \end{array}\right)+\frac{n_{i}\theta }{2}$;
3. randomly choose a sequence from *H*_*i*_; the chosen sequence type is denoted α;
4. for each type β∈*E* for which *P*_αβ_ > 0, compute $\hat{\pi }\left(\beta |H_{i}-\alpha \right)$;
5. compute the quantities *x*_1_ and *x*_2_, where
  $$x_{1}=\theta \hat{\pi }(\beta |H_{i}-\alpha )P_{\beta \alpha }\text{ and }x_{2}=n_{i}^{\left(\alpha \right)}-1.$$
  Then, choose:
  - a coalescence event with probability $\frac{x_{2}}{\left(x_{1}+x_{2}\right)}$;
  - a mutation event (to β) with probability $\frac{x_{1}}{\left(x_{1}+x_{2}\right)}$.
6. depending on the type of event chosen in step 5, we continue as follows:
  - if there is a coalescence event, choose another sequence of type α randomly, and let *n*_*i*−1_: = *n*_*i*_ − 1;
  - if there is a mutation event, mutate the sequence α into a sequence β, without changing *n*_*i*_, i.e., let *n*_*i*−1_: = *n*_*i*_;
7. let *i*: = *i* − 1 and continue until *n*_*i*_ = 1.

After implementing the above algorithm, the coalescence times that are at the core of our method can be deduced. In the genealogy G given in Figure 1, we can deduce the coalescent times from the event times. For example, *T*_7_ = *W*_−1_ whereas *T*_6_ = *W*_−2_ + *W*_−3_ because we have a mutation event before a coalescence event.

#### 3.1.2. Weights of genealogies

After generating genealogies using the Stephens and Donnelly (2000) proposal distribution, it is possible to compute the importance weight *w*^(*j*)^ for each genealogy G^(*j*)^, with *j* = 1, 2, …, *J*. Then *w*^(*j*)^ is given by:

(14) $$w^{\left(j\right)}=\frac{W^{\left(j\right)}}{\sum_{j\;=\;1}^{J}W^{\left(j\right)}},$$

where

(15) $$W^{\left(j\right)}=P(D|G^{\left(j\right)},\theta )\frac{P(G^{\left(j\right)}|\theta )}{Q(G^{\left(j\right)})},$$

with

(16) $$Q(G^{\left(j\right)})=\prod_{i\;=\;0}^{-m}\tilde{q_{\theta }}(H_{i-1}|H_{i}),$$

and

(17) $$P(G^{\left(j\right)}|\theta )=\prod_{i\;=\;0}^{-m}p_{\theta }(H_{i}|H_{i-1}).$$

#### 3.1.3. Estimation of the effective population size

When building genealogies backwards in time, as we move backwards in time, fewer coalescence events occur. As a result, coalescence times close to the present are very short and become larger gradually going back in time. These short coalescence times create an undesirable variability in the estimation of the effective population size. Therefore, we propose to cumulate small coalescence times in order to improve the estimation of the effective population size. These cumulated time intervals are called epochs. To define epochs that get larger as we go backwards in time, we followed (Durbin and Li, 2011), and used a special time scale based on the TMRCA. Forest (2014) adopted the same method.

Finally, we note that the idea of cumulating small coalescence times in order to smooth the graph of the estimator of the effective population size was first proposed by Strimmer and Pybus (2001); it has since become quite standard in the related literature.

Once the genealogies have been simulated using the method described in Section 3.1.1, we cumulate the coalescence times as follows:

- we fix the total number of epochs, *n*_cum_, i.e., the total number of time intervals where we estimate the effective population size;
- for each simulated genealogy G^(*j*)^, we compute the MRCA time, $T_{MRCA}^{\left(j\right)}$;
- we use formula (Equation 18) proposed by Durbin and Li (2011) in order to define epochs where estimates of the effective population size are computed. In other words, the following time cutting points in a genealogy G^(*j*)^, *j* = 1, 2, .., *J* are used:
  (18) $$\begin{array}{l} t_{\text{cut},b}^{\left(j\right)}=0.1\cdot \exp\left(\frac{b}{n_{\text{cum}}}\cdot \log(1+10\cdot T_{MRCA}^{\left(j\right)})\right)-0.1,\\ \;b=1,2,\ldots ,n_{\text{cum}}, \end{array}$$

where $t_{\text{cut},n_{\text{cum}}}^{\left(j\right)}=T_{MRCA}^{\left(j\right)}$.

For each genealogy, formula (Equation 18) gives the boundaries of the epochs, measured from the present to the past where *b* = 0, 1, 2, …*n*_cum_ (in units of *N* generations). The boundaries of epochs are different for each genealogy G^(*j*)^ and depend on the length of the tree. For example if $T_{MRCA}^{\left(1\right)}=1$ in units of *N* generations and *n*_cum_ = 5, then according to Equation (18), the boundaries of the intervals are 0.0615, 0.1609, 0.3215, 0.581 (backward in time). For example, for the first epoch, this means that we must cumulate coalescence times from *T*_*n*_ until reaching 0.0615 *N* generations.

The *skyline plot* can be viewed as a method of moments estimator based on the standard coalescence distributions (Strimmer and Pybus, 2001). For a genealogy G^(*j*)^, we have:

(19) $$E\left(T_{k}^{\left(j\right)}\cdot \left(\begin{array}{c} k\\ 2 \end{array}\right)\right)=N\text{ (generations)},$$

because $T_{k}^{\left(j\right)}$ is exponentially distributed as $\exp\left(\left(\begin{array}{c} k\\ 2 \end{array}\right)\right)$. Therefore, we use the estimate

(20) $$\hat{N}e_{k}^{\left(j\right)}\approx t_{k}^{\left(j\right)}\left(\begin{array}{c} k\\ 2 \end{array}\right),j=1,2,\ldots ,J.$$

The expectation of the accumulated waiting time in order to pass from *n* to ℓ lineages, $T_{n\to \ell }^{\left(j\right)}=\overset{n}{\underset{k=\ell }{\sum }}T_{k}^{\left(j\right)}$, is given by (see, for example, Rodrigo et al., 1999)

(21) $$E\left(T_{n\to \ell }^{\left(j\right)}\right)=\frac{2c}{n(n-c)}N\text{ (generations)},$$

where *c* = *n*−ℓ represents the number of coalesced sequences. From Equation (21), we can see that we can estimate, using the method of moments, the effective population size for the cumulated time of *c* coalescence times by:

(22) $$t_{n\to \ell }^{\left(j\right)}\cdot \frac{n(n-c)}{2c},$$

where $t_{n\to \ell }^{\left(j\right)}=\overset{n}{\underset{k=\ell }{\sum }}t_{k}^{\left(j\right)}$, and *c* = *n*−ℓ. In our case, the cumulated waiting times for each genealogy G^(*j*)^ are deduced from Equation (18): once the boundaries of the intervals of epochs are computed, the cumulated waiting times, $\Delta t_{b}^{\left(j\right)}$ numbered from present to the past, are derived as:

(23) $$\Delta t_{b}^{\left(j\right)}=t_{\text{cut},b}^{\left(j\right)}-t_{\text{cut},b-1}^{\left(j\right)},$$

where *b* = 1, 2, …, *n*_cum_, *j* = 1, 2, …, *J*, and $t_{\text{cut},0}^{\left(j\right)}=0$. It follows from Equations (22, 18) that the estimated effective population size for an epoch *b*, and genealogy G^(*j*)^, *j* = 1, 2, …, *J*, is given by:

(24) $$\hat{N}e_{b}^{\left(j\right)}=\Delta t_{b}^{\left(j\right)}\cdot \frac{d_{b}^{\left(j\right)}\left(d_{b}^{\left(j\right)}-c_{b}^{\left(j\right)}\right)}{2c_{b}^{\left(j\right)}},$$

where $d_{b}^{\left(j\right)}$ is the number of sequences at the beginning of the $\Delta t_{b}^{\left(j\right)}$ interval, and $c_{b}^{\left(j\right)}$ is the number of cumulated coalescence times in the epoch $\Delta t_{b}^{\left(j\right)}$, *b* = 1, 2, …, *n*_cum_, *j* = 1, 2, …, *J*.

The distribution of importance weights of genealogies described by the Equation (15) is an approximation to the posterior distribution *P*(G|D, θ). As a result, one can approximate quantities of interest related to the tree by forming a weighted average of these quantities over the sampled trees as suggested in Stephens (2001).

In our case, we are interested in the estimation of *E*(*Ne*_*b*_), *b* = 1, 2, …, *n*_cum_ from the *J* estimates $\hat{N}e_{b}^{\left(j\right)}$, *j* = 1, 2, …, *J* and we let

(25) $$\begin{array}{l} E\left(Ne_{b}\right)\approx \overset{J}{\underset{j=1}{\sum }}w^{\left(j\right)}\hat{N}e_{b}^{\left(j\right)}. \end{array}$$

In our algorithm, the weighted average of $\hat{N}e_{b}^{\left(j\right)}$ is computed for the same time interval for all *j* = 1, 2, …, *J* that represent the intersections of epochs for the *J* simulated genealogies. This way of proceeding gives us weighted estimates of effective population sizes under the assumption that the effective population size is constant for an epoch. The reason for taking common intervals across genealogies is that $\hat{N}e_{k}^{\left(j\right)}$ estimates the integral (see Pybus et al., 2000)

(26) $${\left(\frac{1}{t_{k}}\int_{v_{k+1}^{\left(j\right)}}^{t_{k}^{\left(j\right)}+v_{k+1}^{\left(j\right)}}\frac{dx}{N_{e}(x)}\right)}^{-1},j=1,2,\ldots ,J.$$

Therefore, to estimate the integral Equation (26) by a weighted average of estimates from *J* genealogies, we must use the same time intervals.

**Figure 2:**
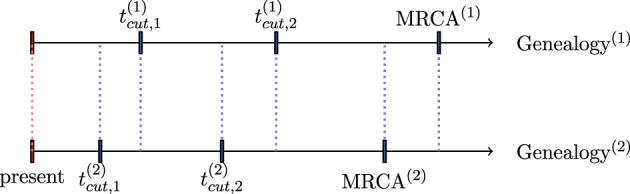
**Division of time axis in the presence of two genealogies**.

For illustration, in Figure 2, we assume that two genealogies G^(1)^ and G^(2)^ are simulated using the method described in Section 3.1.1 with respective weights *w*^(1)^ and *w*^(2)^. Further, we assume that we cumulate coalescence times to obtain *n*_cum_ = 3 epochs. The limits of epochs for a genealogy G^(*j*)^ are denoted $t_{\text{cut},b}^{\left(j\right)},b=1,2$, and the time to the MRCA by TMRCA^(*j*)^, *j* = 1, 2. The detailed calculation of the weighted effective population size per epochs is summarized in the following table:

**Table:** 

**Time interval**	**$\hat{N}e_{\ell },\ell =1,2,\ldots ,2\cdot n_{\text{cum}}$**
$\left[0;t_{\text{cut},1}^{\left(2\right)}\right)$	$w^{\left(1\right)}\hat{N}e_{1}^{\left(1\right)}$ + $w^{\left(2\right)}\hat{N}e_{1}^{\left(2\right)}$
$\left[t_{\text{cut},1}^{\left(2\right)};t_{\text{cut},1}^{\left(1\right)}\right)$	$w^{\left(1\right)}\hat{N}e_{1}^{\left(1\right)}$ + $w^{\left(2\right)}\hat{N}e_{2}^{\left(2\right)}$
$\left[t_{\text{cut},1}^{\left(1\right)};t_{\text{cut},2}^{\left(2\right)}\right)$	$w^{\left(1\right)}\hat{N}e_{2}^{\left(1\right)}$ + $w^{\left(2\right)}\hat{N}e_{2}^{\left(2\right)}$
$\left[t_{\text{cut},2}^{\left(2\right)};t_{\text{cut},2}^{\left(1\right)}\right)$	$w^{\left(1\right)}\hat{N}e_{2}^{\left(1\right)}$ + $w^{\left(2\right)}\hat{N}e_{3}^{\left(2\right)}$
$\left[t_{\text{cut},2}^{\left(1\right)};TMRCA^{\left(2\right)}\right)$	$w^{\left(1\right)}\hat{N}e_{3}^{\left(1\right)}+w^{\left(2\right)}\hat{N}e_{3}^{\left(2\right)}$
[*TMRCA*^(2)^; *TMRCA*^(1)^]	$\hat{N}e_{3}^{\left(1\right)}$

### 3.2. Skywis for heterochronous sampling

**Figure 3:**
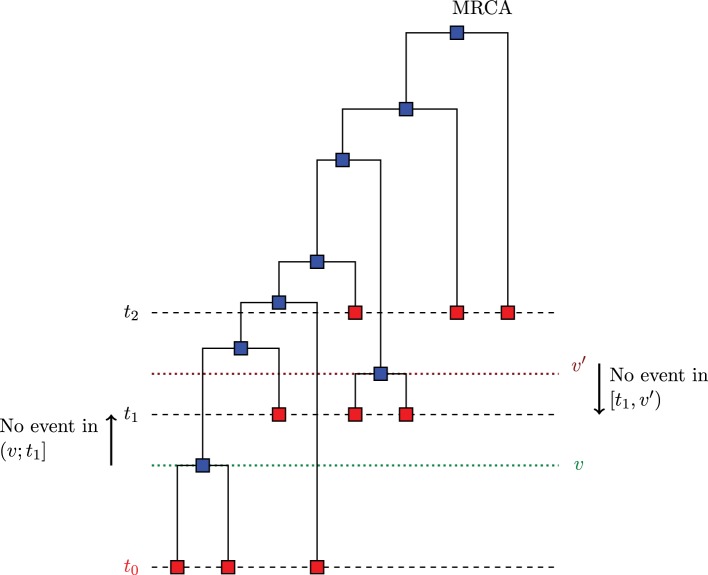
**Example of serially sampled sequences with *S* = 3**. The red squares are the sampled sequences and the blue squares are the sequences derived from coalescence.

The algorithm described in Section 3.1 can be generalized to the case of serially sampled sequences i.e., sequences sampled at different moments in time. Such samples are also called heterochronous. Figure 3 illustrates a case where we sampled sequences at times *t*_0_ < *t*_1_ < *t*_2_, and the time is measured from the present to the past. Let *S* be the number of instants where we sampled sequences (*S* = 3 in Figure 3). Rodrigo and Felsenstein (1999) extend the coalescent likelihood for such heterochronous sequences, a very important issue in the case of rapidly evolving viruses such as HIV. For example, Rodrigo et al. (1999) have estimated, using heterochronous sequences, the viral generation time of HIV type1 (HIV-1). Also, serially sampling rapidly evolving populations is used for dating evolutionary events and divergence times (see e.g., Drummond et al., 2003).

In the presence of serially sampled sequences, we have to adapt the method of Stephens and Donnelly (2000) in order to simulate genealogies in this case. This necessarily involves developing new formulas for the probabilities *p*_θ_(*H*_*i*_|*H*_*i*−1_) and $\tilde{q_{\theta }}\left(H_{i-1}|H_{i}\right)$, as given below.

#### 3.2.1. Backward and forward probabilities, and weights of genealogies

Let *n*^(*s*)^ be the number of additional sampled sequences at time *t*_*s*_, with *s* = 1, 2, …, *S*−1. The main difference between the algorithm for homochronous sequences presented in Section 3.1, and the new algorithm for heterochronous sequences is that the number of lineages increases at the (known) instants *t*_*s*_, *s* = 1, 2, …, *S*−1 where samples of sequences are added. Further, it is necessary to use event times, because the embedded chain differs according to the relative position of these event times with respect to *t*_*s*_, *s* = 0, 1, 2, …, *S* − 1.

In other words, the probabilities *p*_θ_(*H*_*i*_|*H*_*i*−1_) and $\tilde{q_{\theta }}\left(H_{i-1}|H_{i}\right)$ are calculated differently from the case of a single sample of sequences, which has an impact on how the weights of genealogies, *w*^(*j*)^, *j* = 1, 2, …, *J*, are computed.

In order to present our results, we introduce these additional notations:

- *D*_*i, v*_ = {*H*_*i*_, *v*}: represents the set of all sequences present in the population after the *i*^*th*^ event at time *v*; this is a generalization of *H*_*i*_ with the specification of the time of event *i*;
- ε_*s*_: represents the set of all sequences added at time *t*_*s*_;

Our proposal distribution is an adapted version of the Stephens and Donnelly (2000) method for simulating genealogies, to the case of heterochronous sequences. In this case, as mentioned above, we consider that there is a list of pre-specified sampling times *t*_*s*_, *s* = 0, 1, 2…, *S*−1 which are dividing the time axis. In what follows, time is measured from the present to the past and by *event* we mean either a coalescence or a mutation. If an event time *v* is such that *t*_*s*−1_ < *v*<*t*_*s*_ and the time *v*′ of the next event is such that $v^{\prime }>t_{s}$, *v*′ is truncated at *t*_*s*_, i.e., $v^{\prime }\leq t_{s}$. Then, either there is a next event at time $v^{\prime }\leq t_{s}$ or the time is truncated at *t*_*s*_, new sequences are added, and the process starts anew. Thus, from *D*_*i, v*_ one can move to either $D_{i-1,v^{\prime }}=\left\{H_{i-1},v^{\prime }\right\},v<v^{\prime }\leq t_{s},$ where *H*_*i*−1_ is obtained from *H*_*i*_ by a coalescence or a mutation, or to *D*_*i*−1,*t*_*s*__ where *H*_*i*−1_ = *H*_*i*_ + ε_*s*_. In this last case we add ε_*s*_ sequences at time *t*_*s*_ and the process starts anew, with a new set of sequences that includes those at *v*. The moves of the process (embedded chain) are given by the following formulas, and we consider separately the case $v^{\prime }<t_{s}$ and the case $v^{\prime }=t_{s}$.

Case 1: $t_{s-1}<v<v^{\prime }<t_{s}$.

(27) $$\tilde{q_{\theta }}(D_{i-1,v^{\prime }}|D_{i,v})=\{\begin{array}{cc} C^{-1}\frac{\theta }{2}n_{i}^{\left(\alpha \right)}\frac{\hat{\pi }(\beta |H_{i}-\alpha )}{\hat{\pi }(\alpha |H_{i}-\alpha )}P_{\beta \alpha } & \text{ if }M_{\alpha }^{\beta }\\ C^{-1}\left(\begin{array}{c} n_{i}^{\left(\alpha \right)}\\ 2 \end{array}\right)\frac{1}{\hat{\pi }(\alpha |H_{i}-\alpha )} & \text{ if }C_{\alpha }^{\alpha }\\ 0 & \text{otherwise,} \end{array}$$

Case 2: $t_{s-1}<v<t_{s},v^{\prime }=t_{s}$.

(28) $$\tilde{q_{\theta }}(D_{i-1,t_{s}}|D_{i,v})=\{\begin{array}{cc} \text{Pr}(\;\exists \text{ an event in }(v;t_{s}]\;)\cdot C^{-1}\frac{\theta }{2}n_{i}^{\left(\alpha \right)}\frac{\hat{\pi }(\beta |H_{i}-\alpha )}{\hat{\pi }(\alpha |H_{i}-\alpha )}P_{\beta \alpha } & \text{if }M_{\alpha }^{\beta },\\ \text{Pr}(\;\exists \text{ an event in }(v;t_{s}]\;)\cdot C^{-1}\left(\begin{array}{c} n_{i}^{\left(\alpha \right)}\\ 2 \end{array}\right)\frac{1}{\hat{\pi }(\alpha |H_{i}-\alpha )} & \text{if }C_{\alpha }^{\alpha },\\ \text{Pr(no event in }(v,t_{s}]\;) & \text{if }H_{i-1}=H_{i}+{ℰ}_{s},\\ 0 & \text{otherwise}. \end{array}$$

Normally (i.e., in homochronous sampling), the waiting time *W*_−*i*−1_ from the state *D*_*i, v*_ with *t*_*s*−1_ < *v* < *t*_*s*_ to the next event has an exponential distribution with rate λi=(ni2)+niθ2, where *n*_*i*_ is the number of lineages at time *v*. Thus, the probability that there are no events in the interval (*v, v*′]≡(*v, ts*] is given by the survival function

(29) $$Pr(W_{-i-1}>t_{s}-v)=\exp\left(-\lambda_{i}(t_{s}-v)\right),$$

where *W*_−*i*−1_ is the waiting time from state *H*_*i*_ to state *H*_*i*−1_ in a process with homochronous sampling.

In the case of heterochronous sequences, the algorithm for simulating the genealogies backward in time is the following:

1. initialize *n*_*i*_ = *n* and *s* = 0, where *n* is the number of sampled sequences at time *t*_0_ = 0 (present), and *s* is the index of times where we perform the sampling. Further, initialize the cumulated time *t*_cum_ to 0;
2. simulate the time to the next event, *W*_−*i*−1_, as an exponential distribution with parameter $\left(\begin{array}{c} n_{i}\\ 2 \end{array}\right)+\frac{n_{i}\theta }{2}$; let *t*_evt_
  be the observed value;
3. compute $t_{\text{cum}}^{*}:=t_{\text{cum}}^{\left(i\right)}+t_{\text{evt}}$;
4. if $t_{\text{cum}}^{\left(i\right)}<t_{s}$ and $t_{\text{cum}}^{*}>t_{s}$, then
  - let $t_{\text{cum}}^{\left(i-1\right)}=t_{s}$;
  - let $n_{i-1}:=n_{i}+n^{\left(s\right)}$ (add a sample of sequences at time *t*_*s*_);
  - let *s*: = *s*+1 and *i*: = *i*−1, and go to step 2;
  otherwise, go to step 5;
5. let $t_{\text{cum}}^{\left(i-1\right)}:=t_{\text{cum}}^{*}$ and randomly choose a sequence from *n*_*i*_; the chosen sequence type is denoted α;
6. compute the quantities *x*_1_ and *x*_2_, where
  $$x_{1}=\theta \hat{\pi }(\beta |H_{i}-\alpha )P_{\beta \alpha }\text{ and }x_{2}=n_{i}^{\left(\alpha \right)}-1.$$
  Then, choose:
  - a coalescence event with probability $\frac{x_{2}}{\left(x_{1}+x_{2}\right)}$;
  - a mutation event (to β) with probability $\frac{x_{1}}{\left(x_{1}+x_{2}\right)}$.
7. depending on the result in step 6:
  - if there is a coalescence event, choose another sequence of type α randomly, and let *n*_*i*−1_: = *n*_*i*_ − 1;
  - if there is a mutation event, mutate the sequence α into a sequence β, without changing *n*_*i*_, i.e., let *n*_*i*−1_: = *n*_*i*_;
8. let *i*: = *i*−1 and continue until *n*_*i*_ = 1.

After the definition of how to build a genealogy in the case of serially sampled sequences, and the proposal distribution *Q*, we introduce the probability *P* of the genealogy by specifying the probability of passing from the state $D_{i-1,v^{\prime }}=\;\left\{H_{i-1},v^{\prime }\right\}$ to the state *D*_*i, v*_ = {*H*_*i*_, *v*} when there are *n*_*i*−1_ sequences, and we suppose that an event time *v*′ is such that $t_{s}<v^{\prime }<t_{s+1}$ (a coalescence corresponds to a split when viewed from the past to the present). Therefore, as for the backward transition probabilities, we consider separately the case *v*>*t*_*s*_ and the case *v* = *t*_*s*_.

Case 1: $t_{s}<v<v^{\prime }<t_{s+1}$.

(30) $$p_{\theta }(D_{i,v}|D_{i-1,v^{\prime }})=\{\begin{array}{cc} \frac{n_{i-1}^{\left(\alpha \right)}}{n_{i-1}}\frac{\theta }{\left(n_{i-1}-1+\theta \right)}P_{\alpha \beta } & \text{if }M_{\alpha }^{\beta }\\ \frac{n_{i-1}^{\left(\alpha \right)}}{n_{i-1}}\frac{n_{i-1}-1}{n_{i-1}-1+\theta } & \text{if }C_{\alpha }^{\alpha }\\ 0 & \text{otherwise}. \end{array}$$

Case 2: $t_{s}<v^{\prime }<t_{s+1}$ and *v* = *t*_*s*_.

(31) $$p_{\theta }(D_{i,v}|D_{i-1,v^{\prime }})=\{\begin{array}{cc} \text{Pr( }\exists \text{ an event in }[t_{s};v^{\prime })\;)\cdot \frac{n_{i-1}^{\left(\alpha \right)}}{n_{i-1}}\frac{\theta }{\left(n_{i-1}-1+\theta \right)}P_{\alpha \beta } & \text{if }M_{\alpha }^{\beta }\\ \text{Pr( }\exists \text{ an event in }[t_{s};v^{\prime })\;)\cdot \frac{n_{i-1}^{\left(\alpha \right)}}{n_{i-1}}\frac{n_{i-1}-1}{n_{i-1}-1+\theta } & \text{if }C_{\alpha }^{\alpha }\\ \text{Pr( no event in }[t_{s},v^{\prime })) & \text{ if }H_{i}=H_{i-1}-{ℰ}_{s}\\ 0 & \text{otherwise}, \end{array}$$

where:

- the probability that there are no events in the interval $\left[t_{s},v^{\prime }\right)$ is given by:
  (32) $$\mathrm{Pr}(Wt_{-i-1}>v^{\prime }-t_{s})=\exp\left(-\lambda_{i}(v^{\prime }-t_{s})\right).$$
- $n_{i-1}^{\left(\alpha \right)}$ represents the number of sequences of type α in $D_{i-1,v^{\prime }}=\left\{H_{i-1},v^{\prime }\right\}$;
- *H*_*i*_ = *H*_*i*−1_ − ε_*s*_: represents the event of adding the set of sequences ε_*s*_ at time *t*_*s*_.

As in the case of homochronous sequences, after computing the probabilities $p_{\theta }\left(D_{i,v}|D_{i-1,v^{\prime }}\right)$, and $\tilde{q_{\theta }}\left(D_{i-1,v^{\prime }}|D_{i,v}\right)$ for a genealogy *G*^(*j*)^, *j* = 1, 2, …, *J*, the importance weights may be derived from Equations (14–17).

#### 3.2.2. Estimation of the effective population size for heterochronous sequences

For heterochronous sequences, the method for producing a *skywis plot* is similar to the one defined in Section 3.1.3. The main difference lies in the definition of epochs in this case ^1^. In the presence of *S* serially sampled sequences, we cumulate the coalescence times as follows:

- for each simulated genealogy G^(*j*)^, we compute the MRCA time, $T_{MRCA}^{\left(j\right)}$, *j* = 1, 2, …, *J*;
- we fix the number of epochs at $n_{\text{cum}}^{\left(s\right)}$ in each time interval (*t*_*s*_; *t*_*s*−1_) where no new sample is added, *s* = 1, 2, …, *S*, $t_{S}=T_{MRCA}^{\left(j\right)}$, and *t*_0_ = 0 (present);
- in order to define the epochs, the time cutting points in a genealogy G^(*j*)^, *j* = 1, 2, .., *J* are computed as follows:
  (33) $$t_{\text{cut},b}^{\left(j,s\right)}=t_{s-1}+0.1\cdot \exp\left(\frac{b}{n_{\text{cum}}}\cdot \log(1+10\cdot t_{s})\right)-0.1,$$

where $b=1,2,\ldots ,n_{\text{cum}}^{\left(s\right)}$ and *s* = 1, 2, …, *S* − 1.

For each genealogy and for each time interval (*t*_*s*_; *t*_*s*−1_), *s* = 1, 2, …, *S*, formula Equation (33) gives the limits of the epochs from the present to the past in units of *N* generations.

In practice, we performed minor smoothing at times *t*_*s*_, because the addition of new sequences creates an artificial discontinuity at *t*_*s*_, *s* = 1, 2, …, *S*. Therefore, the population size in the first epoch after *t*_*s*_ is set to be equal to the effective population size in the epoch preceding the addition of new sequences.

## 4. Results

To test the ability of our method to capture the demographic signal contained in the DNA sequences, we simulated several demographics scenarios. Further, we compared the results of the *skywis plot* with those of the *generalized skyline plot* that uses single tree, and the *Bayesian skyline plot* that uses MCMC approach. These methods are the closest to our approach.

The DNA sequences were simulated using the *fastsimcoal* program (Excoffier and Foll, 2011) which allows us to consider several demographic scenarios and different mutation models. The genealogies were simulated ^2^ using the method described in Section 3.1.1. In all our simulations, the coalescence times were cumulated into $n_{\text{cum}}=\sqrt{n}-1$ epochs according to the method described in Section 3.1.3, where *n* represents the number of simulated DNA sequences. After that, we derive the *skywis plot* using Equations (24, 25).

The *generalized skyline plot* was performed as follows:

1. From the DNA sequences generated by *fastsimcoal*, we estimated a phylogeny using the PHYLIP program (the PHYLogeny Inference Package Felsenstein, 1989) using the maximum likelihood method with a molecular clock constraint (we used the *dnamlk* program).
2. Based on the estimated tree produced by PHYLIP, we used the APE package (Paradis et al., 2004) to produce the *generalized skyline plot* according to the optimal strategy for grouping adjacent coalescent intervals introduced by Strimmer and Pybus (2001).

The *Bayesian skyline plot* was performed using the BEAST program, version 1.8.1. In order to reproduce a parametrization which is as close as possible to ours, we used (Hasegawa et al., 1985) substitution model with equal base frequencies, and a strict clock with rate 1.

Below, we present our results according to the demographic models we considered.

### 4.1. Constant effective population size

In this case, we consider 50 simulated DNA sequences with parameters:

- number of nucleotides: 10,000;
- constant effective population size: 2000 generations;
- no recombination and no population structure;
- mutation rate equals to 2·10^−7^: therefore (θ = 8);
- JC69 (Jukes and Cantor, 1969) finite sites model.

**Figure 4:**
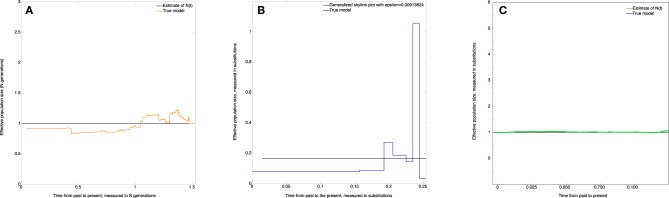
**Constant effective population size. (A)** *skywis plot*, **(B)** *generalized skyline plot*, **(C)** *Bayesian skyline plot*.

The estimate of the effective population size (*skywis plot*) is shown in Figure 4A. We observe that the *skywis plot* (orange line) gives a relatively smooth curve of the effective population size. Further, the estimation turns around the real value *N*, with a slight over-estimation close to the present, which can be explained by the fact that when the mutation rate θ is large, the sampled sequences are all different, and we have many mutations before one coalescence; thus, coalescence times are longer, and the corresponding population sizes are larger (see Section Simulation of Genealogies.)

In Figure 4B we present the *generalized skyline plot* (in substitution units). In this case, the form of the graph is not recognizable as a constant line.

The *Bayesian skyline plot* is given in Figure 4C. In this case, the graph is very smooth and is easily recognizable as a constant line.

### 4.2. Piecewise constant function

**Figure 5:**
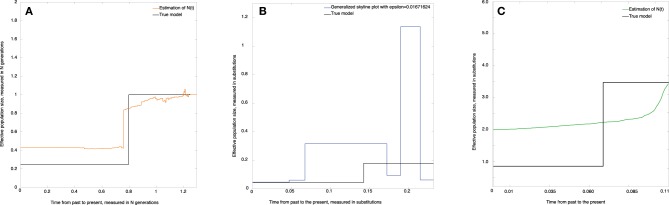
**Skywis plot for data simulated from the population model where *N*(*t*) = 10, 000, if *t* < 5000 generations, and *N*(*t*) = 2500 otherwise (time from the past to the present)**. **(A)** *skywis plot*, **(B)** *generalized skyline plot*, **(C)** *Bayesian skyline plot*.

In this section, we present results where 25 DNA sequences of length 10,000 nucleotides and mutation rate μ = 5·10^−4^ were simulated under the JC69 mutation model. We assume that the effective population size follows the piecewise constant model function:

(34) $$N_{e}(t)=\{\begin{array}{ll} N & \text{ if }t<x\\ aN & \text{otherwise,} \end{array}$$

where *N* = *N*(0) = 10^4^, *x* = 5000 *generations, a* = 0.25 (see Figure 5), and the time *t* is measured from present to the past.

Figure 5A represents the non-parametric estimate (*skywis plot*) of the effective population size for a number of epochs equal to *n*_cum_ = 4. We note that the *skywis plot* was able to detect well enough the change-point of the size of the actual population which dates back 5000 generations. However, the *skywis plot* seems to overestimate the effective population size for *t* > 5000 generations.

In Figure 5B we present the *generalized skyline plot*. The *skywis plot* gives a better result than the *generalized skyline plot* close to the present, while the estimate given by the *generalized skyline plot* is closer to the true value when we approach the MRCA.

The *Bayesian skyline plot* presented in Figure 5C is very smooth and generally reproduces the true history except closer to the present, where the Bayesian skyline plot over-smoothes the effective population size.

### 4.3. Exponential population growth

In this section, we suppose that the effective population growth is exponential assuming an instantaneous growth rate that is proportional to the current population size according to the equation *N*_*e*_(*t*) = *N*exp(−β*t*) from present to the past.

Using the *fastsimcoal* program, we simulate 50 DNA sequences with the following parameters:

- Number of nucleotides: 1000;
- *N* = *N*_*e*_(0) at time *t* = 0: 10,000;
- no recombination, and no population structure;
- mutation rate: 5·10^−7^ (θ = 1);
- JC69 finite sites model;
- β = 1 (in generations).

**Figure 6:**
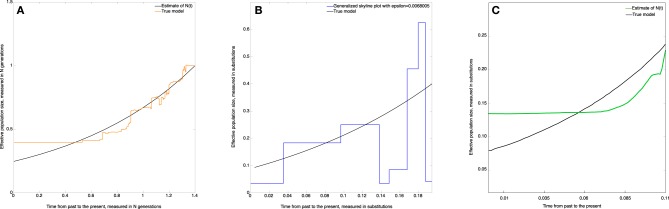
***Skywis plot* for DNA sequences simulated from an exponential model with β = 1. (A)** *skywis plot*, **(B)** *generalized skyline plot*, **(C)** *Bayesian skyline plot*.

The *skywis plot* for the simulated DNA sequences from the exponential model described above is given in Figure 6A.

The result given in Figure 6A is quite good in the sense that the size of the effective population decreases steadily from the present to the past and follows the exponential curve quite closely most of the time. However, we note that the estimated effective population size is almost constant from some point in time when approaching the TMRCA. This is explained by the fact that for the last two sequences the theoretical average time to coalesce represents half the length of the tree, and from this point in time there is no much variability in the estimate of the population size. In particular, this remark led us to consider heterochronous sampling in order to improve the effective population size estimate.

In Figure 6B the time is measured in substitution units and we present the *generalized skyline plot*. As before, the *generalized skyline plot* has a fluctuating shape but it exhibits a certain tendency to decrease toward the past. In the end, when we approach the time of the MRCA, the *generalized skyline plot* gives an estimate that is close to the true value.

In Figure 6C, we present the *Bayesian skyline plot*. As in the other scenarios, the *Bayesian skyline plot* produces a very smooth curve; in this case it suggests that the population had a mild exponential expansion. However, we note that the curve remains constant closer to the MRCA.

### 4.4. Exponential population growth and heterochronous sequences

In order to test the methodology proposed in Section 3.2, we use the same parameters as in Section 4.3, but by assume that the 50 sequences were collected at different moments in time such as:

- *n*_0_ = 25 (present);
- *n*_1_ = 15 at time *t*_1_ = 0.5 in units of *N* generations (measured from present to the past);
- *n*_2_ = 10 at time *t*_1_ = 1 (*N* generations).

**Figure 7:**
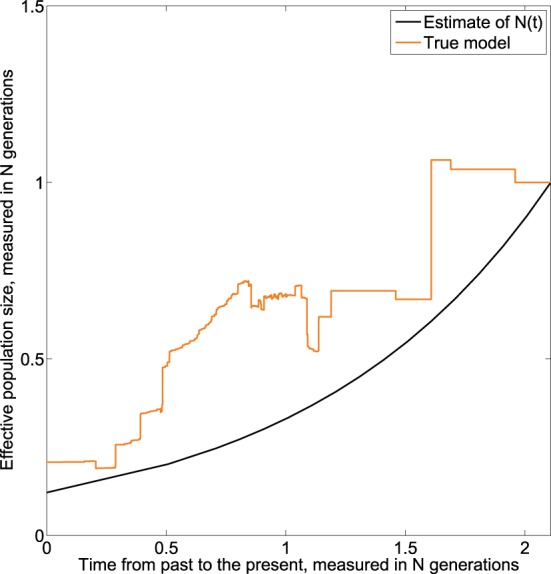
**Skywis plot for DNA sequences simulated from an exponential model with 3 serial samples at times *t*_0_ = 0, *t*_1_ = 0.5, *t*_1_ = 1 (in units of *N* generations) from the present to the past, and β = 1 generations**.

The result given in Figure 7 suggests that the effective population decreases exponentially from present to the past. Further, we note that the estimated effective population size continues to decrease when approaching the time of the MRCA, which is a net improvement over the homochronous case. This could be explained by the fact that as more sequences are added over time, more information is available as one approaches the MRCA.

## 5. Discussion

The *skywis plot* is a new flexible method for exploring the demographic history of a sample of DNA sequences based on coalescent theory. Our nonparametric method is likelihood-based and uses IS. More precisely, we generate a large number of genealogies, both their times and their topology; further, we use the importance weights of these genealogies to compute a weighted average of the effective population size per epoch. This allows us to produce estimates that exhibit clear cut population growth tendencies over time, which is the main purpose of this approach, given that it is nonparametric. In practice, we expect our method to be used as a preliminary procedure that could be supplemented by a parametric analysis.

We present a framework of the new method and test by simulation its ability to capture the demographic signal contained in the DNA sequences under several demographic scenarios. Moreover, we consider both homochronous and heterochronous data using a simple substitution model, JC69 (Jukes and Cantor, 1969). We could also have considered more complicated substitution models, except those that allow variation in evolutionary rates among lineages.

For illustration we present the results given by the generalized skyline plot that uses a single genealogy, and those obtained by the *Bayesian skyline plot* that uses an MCMC approach. Although the *Bayesian skyline plot* is smoother than the *skywis plot*, our estimator is able to capture the shape of the effective population size Ne(t), as well as its main change points, but in some examples it had a (slight) tendency to overestimate the population size as we approached the MRCA. This is not surprising, given that the simulation our estimation method entails first setting a constant population size (where coalescence times are longer) and further operating an adjustment through a weighting system. Further, note that, unlike the methods based on a single tree, it is possible to extend the *skywis plot* and include recombination. Indeed, recombination induces a graph structure rather than a tree, and IS methods in this context already exist (e.g., Fearnhead and Donnelly, 2001).

As a future development, we expect the method to be improved by considering an iterative procedure, in which the present approach would be the first estimation step. As a new approach, the *skywis plot* remains to be tested on more complex demographic models, and models of substitution that could be more realistic, especially for rapidly evolving RNA viruses. Also, the *skywis plot* can be easily extended to include multilocus data because, when there is no recombination, the same IS scheme can be applied.

## Funding

The Ph.D. studies of SA were supported in part by scholarships awarded by the ISM (Institut des Sciences Mathématiques) and stipends out of NSERC (Natural Sciences and Engineering Research Council) research grants.

### Conflict of interest statement

The authors declare that the research was conducted in the absence of any commercial or financial relationships that could be construed as a potential conflict of interest.

## Footnotes

^1^The reason we changed the way we define the epochs is that the number of sequences rises at the instants of the serial sampling, so the method used in Section Simulation of Genealogies is not appropriate.

^2^The simulation of the genealogies was performed using MATLAB programming language (MATLAB, 2013) and the *Parallel Computing Toolbox* which allows parallelization of the simulation of genealogies. This is possible when using IS.
